# Greek Medicine Practice at Ancient Rome: The Physician Molecularist Asclepiades

**DOI:** 10.3390/medicines4040092

**Published:** 2017-12-12

**Authors:** Luigi Santacroce, Lucrezia Bottalico, Ioannis Alexandros Charitos

**Affiliations:** 1 Ionian Department, University of Bari “A. Moro”, Piazza Umberto I, 70121 Bari, Italy; bottalico.lu@gmail.com; 2 Policlinico University Hospital, P.zza G. Gesare 11, 70124 Bari, Italy; alexanestesia@hotmail.com

**Keywords:** Asclepiades, Hellenistic medicine, atomism, medical philosophy

## Abstract

**Background:** In the pre-Hellenistic period, the concept of medicine was not well-defined. Usually, a disease was considered as a divine punishment and its treatment was devolved to the priests who asked for healing from the divinities. The only job that could be compared to medical practice was a kind of itinerant medicine, derived from the Egyptian therapeutic tradition based only on practical experience and performed by people that knew a number of remedies, mostly vegetable, but without any theoretical bases about the possible mechanisms of action. Opinions about the human nature (naturalistic thinking) and the origin of the illness and heal were the basis of Greek medicine practiced by ancient priests of Asclepius. However, with the evolution of the thought for the continuous research of “κόσμος” (world) knowledge, philosophy woulld become an integral part of medicine and its evolution. This close relationship between philosophy and medicine is confirmed by the Greek physician Galen in the era of the Roman Empire. **Methods:** Philosophical thought looked for world knowledge starting from mathematics, physics, astronomy, chemistry, medicine, psychology, metaphysics, sociology, and ethics. We must keep in mind that, according to the ancient people, the physicians could not heal the patients without the aid of a “divine God” until medicine, thanks to the Hippocratic practice, became more independent from the supernatural, and contemporary, ethical, and professional. Many physicians were philosophers, as confirmed by their views of life, such as Hippocrates of Cos, Aristotle (hailed as the father of comparative anatomy and physiology), Pythagoras of Samos, Alcmaeon of Croton, Empedocles, Praxagoras, Erasistratus, Galen, and others, including Asclepiades of Bithynia (atomists affinity). Asclepiades, a Greek physician born in Prusa, studied in Athens and Alexandria. His thought was influenced by Democritus’ theories, refusing extensively the Hippocratic ideas that diseases are a result of mood imbalance. **Results:** Differing from the current Hippocratic idea, only in extreme cases he prescribed medications and bloodletting, two of the most-used therapies of that time. He usually prescribed therapies based on the Epicurean thought, then consisting of walks and music, massages, and thermal baths. He anticipated the modern idea of the body consisting of atoms, and believed that between the atoms exist empty spaces called pores. As the founder of the so called *Methodist School*, he was the first to divide acute and chronic diseases, and thought that body weakness was dependent on the excessive width of the pores, while their excessive shrinkage determines fever. According to his student Caelius Aurelianus he was the first to adopt tracheotomy as an emergency therapy for diphtheria. **Conclusions:** Although it is very difficult to reconstruct the theories of Asclepiades of Bithynia because of the lack of original texts, this paper attempts to focus his role and his thought in affirming the Greek medical practice in ancient Rome and to highlight his modernity.

## 1. The Appearance of Greek Medicine at Ancient Rome

Philosophy is inextricably related to the societies evolution. In the beginning, the Roman attitude toward medicine was almost simple. We must not forget that the Roman civilization was hardly satisfied with abstract concepts, was predominantly pragmatic, realistic and had its own perceptions of life and the human being [1,2,3,4]. For the Romans, a human is mainly a “*cives*” (citizen), a social entity with legal hypostasis, a concept on which the whole of Roman civilization was built [5,6,7]. In Rome, medicine was often practiced within the family by the pater familias (or, according to a number of evidences, by old women), but without basic theories and mainly using vegetables and magic formulas, thus proving to be an empirical science [8]. After a time appeared the “medicus” (physician), usually a slave or a “*libertus*”, considered a useful craftsman. The best of them were awarded and educated in medicine by their owners to keep them as personal or family doctors.

A real education in the art of medicine did not exist in Rome up to the 1st–2nd century C.E., so anyone could declare himself a doctor and open a “clinic” without any theoretical knowledge and practical experience. For this reason Galen, in the 2nd century C.E., reports that many of his alleged “colleagues” did not know how to read a text [9,10].

At the beginning of the Roman age, the number of known diseases was limited and primarily affected the respiratory and gastrointestinal tracts, with a large prevalence of infectious diseases favored by bad hygienic conditions [11]. Nowadays, thanks to a number of pathocenosis studies, we know that the number of diseases affecting the Romans was not only 50–60 as reported by Galen and other authors of the time, but much more numerous and “modern”, especially eye and gynecological diseases, as well as dermatological and rheumatologic. Many cases of oncological pathology, in particular of the bone, have also been demonstrated, so we can assume that tumors of parenchymatous organs were relatively high [12,13,14,15].

We must mention that in the military they had developed a rapid trauma aid system, improved afterwards by Galen. After contact with Southern Italy (Magna Graecia), the peninsula of Greece and Asia Minor, and Hellenistic Egypt (here we must mention the important Greek medical school of Alexandria by Herophilus and Erasistratus), would start to become acquainted with the extraordinary Greek thought and its art, philosophy and, of course, medicine. Pliny reports the name of the first Greek physician around 218 B.C.E., Archagathos of Sparta, who, after 600 years of no physician existing in Rome [16], introduced Greek medicine.

Thus, after the conquest of the peoples of the Eastern Mediterranean, the Hellenistic medical world spread its knowledge to the Roman people and medicine in the Roman Empire began to evolve.

As in various cities of the Greek world, various medical school doctrines had also been founded by the Greek physicians in Rome, as the “*Dogmatiki*” (o “*Pneumatiki*”) by Athenaeus of Attalia, which added the concept of the “pneuma” (πνεύμα) to the Hippocratic “humoral” theory. According to this idea, a sort of thing between the body and the soul, whose life depended from its union with them. When this was inhaled with infected air, it caused a disease.

Another one was the *“Empiriki” school*, founded from Serapion of Alexandria and Philinus of Kos, whose names derive from their knowledge about medicine stemming from their clinic experiences [5,17,18,19].

Greek medicine was finally established in Rome from 91 B.C.E. by the Bithynian doctor Asclepiades (Ασκλεπιάδης, 130 B.C.E.–40 B.C.E.) who was, at first, a rhetorical teacher and later a physician, and friend of Cicero. He studied in Athens and learned the art of medicine at Alexandria of Egypt, practicing medicine in Greece and Asia Minor before moving to Rome [20].

As reported by various sources, and in a synthetically exhaustive manner by Perilli, Asclepiades was strongly attacked by Menodotus Nicomediae, attested to completely different ideological positions, which was, in turn, attacked by Galen. These exchanges of cross-accusations allow us to draw a series of indirect information on the thinking behind the medicine in the view of the three contenders [21].

He brought a prestige to Greek medicine and, finally, laid the foundations for the *“Methodiki”* school, which significantly influenced medicine. The main guideline of this Methodist school was to observe some general symptoms of the patient as a priority to decide on proper therapy.

His medical practice was very successful, so he was also mentioned by Galen (164 C.E.). He advocated that the duty of the physician is to deal safely, quickly, and pleasantly. He was the first to distinguish acute and chronic diseases and skillfully practiced surgery. He especially focused on mental illnesses with new interpretations and treatments, and distinguished the diseases in *status strictus* (constricted), *status laxus* (relaxed), and *status mixtus* (mixed). A supporter of a hygienic lifestyle, he was an opponent of the Hippocratic “*perception*” concept, abandoning the Hippocratic humoral theory. Antiochus of Ascalon describes Asclepiades “*second to none in the art of medicine and acquainted with philosophy, too*” [22,23,24,25].

## 2. Medicine from Atomism to Molecular Theory

The philosophical thought of Asclepiades is very close to Democritus through the Epicurean philosophical school of Athens, and seems that he also supported the original principle of the matter conception expressed three centuries before by Heraclides of Pontus (388–310 B.C.E.). Indeed, Heraclides proposed a corpuscular theory that the world was created by various combinations of atoms, using the term “*ὂγκoι*” (masses), where they are always in motion. These forms and mathematically measurable movements give rise to all the natural phenomena. However, Heraclides, which had an affinity to Plato, in his works also mentioned supernatural events like *mantic dreams* and *healing therapy* through the divine Asclepios incubation, as other physicians did later, e.g., Galen.

Plato does not consider the theory of Democritus, and in some of his dialogues he proves it. Asclepiades certainly was not Platonic, but has an Epicurean affinity [26].

The application of atomism to physiology enabled the Democritean school to formulate a highly consistent mechanistic view of medicine. His atomistic theory ruled that illness was caused by an imbalance of atoms (*ἂτoμα*) according to which life phenomena are subject to mechanical laws.

In his teachings he also gave a new look to the moral positions, which he radically changed, and explained, human, biological, and psychological phenomena. He accepted that the being is an indivisible matter, an atom, that is not intersecting. His body must be viewed in a purely mechanical way, but his soul is made up of a very thin matter or, more correctly, a result of the movement of this matter. The whole world is likened alive, having a soul, the divine, which, in turn, is derived from the moving matter.

The senses are explained by physical contacts. The logical knowledge of good, which puts the peace of the soul and its harmony over pleasure and pain, supports Democritus and his morality, establishing a high euphemism based on the moral autonomy of reason [27,28,29,30].

Later, Democritus inspired the philosophy of Epicurus and Lucretius. Epicurus (341–270 B.C.E.) founded his own philosophical school, named “*Garden of Epicurus*”, which is considered to be one of the most famous schools of Greek philosophy. The basic principles of his teaching are as follows:

(a) the universe is perennial, infinite and the variations of the events in this eventually occur based on the movements and interactions of the corpuscular movements in the empty space;
(b) the gods do not reward, punish or help humans;
(c) with the end of the life becomes the end not only of the body, but of the soul, because it is made of atoms, as well as any other part of the body; and
(d) for humans pleasure is the primary good.

About the pleasure of humans as the primary good, this is another difference from Plato which considered it as minor.

Epicureanism became one of the principal philosophies of the Roman world. It was primarily an ethical thinking, and in ancient medical history the philosophy needed an explanatory basis for the physical world, and for this purpose Epicurus selected the atomism of Democritus, albeit with an important reservation: research. His school formed an idealistic community of disciples.

Epicurean philosophy left open the phenomenon of randomness as opposed to the deterministic and teleological demands of the Platonists and Aristotelians, as opposed to the use of Democritus’ necessity.

This atheistic approach is of particular interest, and he considers the matter that even the soul is the accidental movement of the matter from orbit to orbit, which also creates diversity in nature—an impressive quantum approach.

Epicurus supports his physical theory in the physics of Leucippus and Democritus, which he completed in Aristotle’s biological ethics, creating a coherent ontology.

According to the corpuscular theory, the original elements are individuals and around them there was the empty space. Atoms are the smallest pieces of matter, invisible and elusive, perceived only by their intellect, solid, without any gaps in them. They cannot be sliced or broken up or destroyed. They are anarchic and eternal and they remain unchanged in unions and separations.

These are the elements who move and join in inexhaustible combinations and create the material world. According to Epicurus the physiology has a practical character for human life. Scientific research is not an end in itself, but a means of pursuing bliss, based on the common sense, interpretation of nature and natural phenomena, and their effect on human life.

Knowledge of the causes and functioning of natural phenomena does not have to go deeper into exploring the issues, as well as the exhaustive research on these issues, but they are necessary, despite their relative nature, to eliminate fear and anxiety (mythical, religious, metaphysical perceptions, etc.) about the nature and function of the world and about their effect on human life and action.

An important presentation of Epicurean philosophy has survived by the Latin poem *“De rerum natura”* by Lucretius (96–55 B.C.E.). This poem is of particular interest to science historians because it is one of the few extended scientific texts which has come to us intact, and, although its fortunes have varied as the fortunes of physical science have fluctuated, and it represents an uninterrupted scientific tradition over a period of twenty centuries [31,32].

Asclepiades overcame the individual theory of Democritus and Epicurus for the medical thought, which had him as the starting point: the world has been created by various combinations of atoms, the molecules (*μέρη*) that create the forms, and their mathematically-measurable movements give birth to all natural phenomena.

Thus, Ascelpiades puts the foundations of molecular concept into medicine, starting from the theories of atomists. Argued that the human body is formed by tubes and pores (*Πόροι*), else narrower and elsewhere larger, made of combinations of atoms, and that other bodies were constantly moving within this system, with gender differences [33]. According to Ascelpiades health coincides with the uninterrupted flow of these corpuscular forms, while its delay or interruption, for whatever reason, gives rise to disease [34,35].

The Asclepiades conceptions after his death continued to be spread thanks to his student *Themison* of *Laodicea* in the time of Pompeii (1st century C.E.), who is considered the real founder of the *“Methodiki”* school, as opposed to the other two main schools of the time: “Dogmatiki” (or “Pneumatiki”) and “Empiriki”.

Later, Agathinus of Sparta, a member of the Dogmatiki school, founded in Rome the *“Eklektiki”* medical school that combined the Empiriki perceptions with the Methodiki, not abandoning the teachings of the Dogmatiki.

Two hundred years later, the more traditionalist Galen and Caelius Aurelianus did not agree with the thought of Ascelpiades about corpuscular theory and his atheistic approach [36,37].

Regarding therapy, Asclepiades always claimed: “*drink, food and enema*” and advocated diet, exercises, thermal treatments, cold baths, and drinking wine as principal therapeutics. We know other various treatments with many variation of drugs thanks to physicians like Galen, Celsus, Largus, and others. Pliny refers about a medical prescription of the *oxymel*, an antidote which is the combination of “*ten minae of honey, five heminae of old vinegar, a pound and one fourth by weight of salt from the sea, added to five sextarii of water*”. Pliny refer that Asclepiades “*admits that oxymel was beneficial against the snake known as Seps as well as against poisoning by opium and mistletoe*”. Another drug for the cough, from the many formulas of Ascelpiades, is available through a Galen’s manuscripts: “*Pontic rhubarb, Cilician crocus saffron, opium, frankincense, myrrh, Celtic nard, and storax all administered with honey mixed with wine*” [38].

Furthermore, Asclepiades believed that arts, and moreover music, give mental balance and, therefore, he used these to treat mentally ill patients. His passion for surgery will bring him to perform the first elective tracheostomy for the treatment of upper airway obstruction due to pharyngeal inflammation, probably for tonsillar obstruction or diphtheria, but there is no agreement on this [20,39,40].

## 3. Conclusions

Asclepiades practiced medicine openly opposing to the current Hippocrates’ thought, stating that the only therapy should consist of hygienic and good nutritional habitudes, and a relaxed life. These prescription were targeted to restore the equilibrium between atoms passing the body and to preserve their reciprocal distance, reserving appropriate medication to selected, extreme conditions only.

Thanks to Asclepiades the medical art began to be considered a profession and a highly-valued scientific job, inducing a great interest in Romans. Due to this interest, the emperor Caius Julius Caesar intended decree that “…*Omnisque medicinam Romae professos et liberalium artium doctores, quo libentius et ipsi urbem incolerent et ceteri adpeterent, civitate donavit*” to grant Roman citizenship to any doctor practicing in Rome, and also instituted the formal schools of medicine. His successor Caesar Augustus, confirmed this law and instituted the “Valetudinaria”, military hospitals along the borders of the Roman empire [41].

Based on such considerations, modern medicine must thank Asclepiades for his lessons about the importance of the hygiene to avoid infection and to maintain a good health status, the moderate use of drug therapy and only for specific conditions, and the importance of correct nutrition. However, we also have to remember his primordial vision of the body by a “biochemical” point of view, because of his atomistic and molecular theory and, most important for clinical practice, the ability to empathize with patients to improve their health status and therapeutic adherence [42,43].

In the end, we can consider Asclepiades the forerunner of the modern clinician, who must have a good basic and clinical knowledge to identify a clinical condition and its causes, aimed to prescribe a personalized therapy.
